# Supplementary material for: Integrating genomic structural equation modeling and experimental validation to unravel the genetic basis of male genital lichen sclerosus
Source: Front Immunol. 2026 Jul 16;17:1857945. doi: 10.3389/fimmu.2026.1857945 (PMC13435250; doi:10.3389/fimmu.2026.1857945)
Supplement: Supplementary file 6 [file SupplementaryFile5.zip › result/fuma_PSYCH_factor_sCCA1_focus.chr9.chr9.33578334.34642243.pop1.pdf]

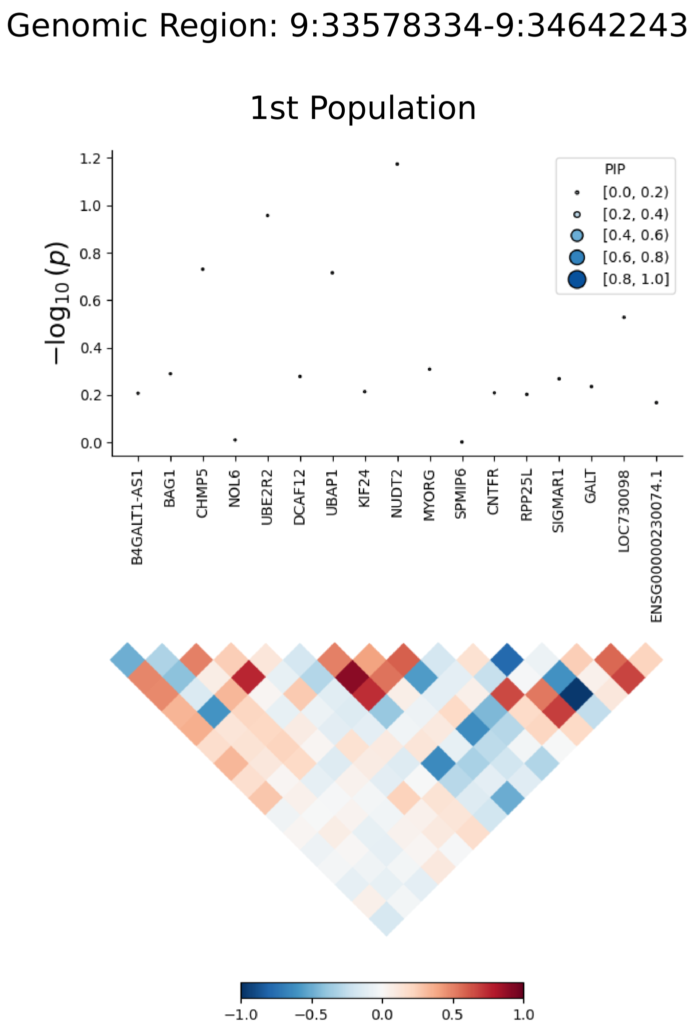
Genomic Region: 9:33578334-9:34642243
1st Population
PIP
[0.0, 0.2)
[0.2, 0.4)
[0.4, 0.6)
[0.6, 0.8)
[0.8, 1.0]
$-\log_{10}(p)$
1.2
1.0
0.8
0.6
0.4
0.2
0.0
B4GALT1-AS1
BAG1
CHMP5
NOL6
UBE2R2
DCAF12
UBAP1
KIF24
NUDT2
MYORG
SPMIP6
CNTFR
RPP25L
SIGMAR1
GALT
LOC730098
ENSG00000230074.1
−1.0
−0.5
0.0
0.5
1.0
